# Expansion of Parasite-Specific CD4^+^ and CD8^+^ T Cells Expressing IL-10 Superfamily Cytokine Members and Their Regulation in Human Lymphatic Filariasis

**DOI:** 10.1371/journal.pntd.0002762

**Published:** 2014-04-03

**Authors:** Rajamanickam Anuradha, Parakkal Jovvian George, Luke E. Hanna, Paul Kumaran, Vedachalam Chandrasekaran, Thomas B. Nutman, Subash Babu

**Affiliations:** 1 National Institutes of Health—International Center for Excellence in Research, Chennai, India; 2 National Institute for Research in Tuberculosis, Chennai, India; 3 Laboratory of Parasitic Diseases, National Institutes of Allergy and Infectious Diseases, National Institutes of Health, Bethesda, Maryland, United States of America; University Clinic Bonn, Germany

## Abstract

**Background:**

Lymphatic filariasis (LF) is known to be associated with an increased production of IL-10. The role of the other IL-10 family members in the pathogenesis of infection and/or disease is not known.

**Methodology/Principal Findings:**

We examined the expression patterns of IL-10 family members – IL-19, IL-24 and IL-26 in LF. We demonstrate that both CD4^+^ and CD8^+^ T cells express IL-19, IL-24 and IL-26 and that the frequency of CD4^+^ T cells expressing IL-19 and IL-24 (as well as IL-10) is significantly increased at baseline and following filarial antigen stimulation in patients with LF in comparison to individuals with filarial lymphedema and uninfected individuals. This CD4^+^ T cell expression pattern was associated with increased production of IL-19 and IL-24 by filarial – antigen stimulated PBMC. Moreover, the frequency of CD4^+^ and CD8^+^ T cells expressing IL-26 was significantly increased following filarial antigen stimulation in filarial lymphedema individuals. Interestingly, IL-10 blockade resulted in diminished frequencies of IL-19^+^ and IL-24^+^ T cells, whereas the addition of recombinant IL-10 resulted in significantly increased frequency of IL-19^+^ and IL-24^+^ T cells as well as significantly up regulated IL-19 and IL-24 gene expression, suggesting that IL-10 regulates IL-19 and IL-24 expression in T cells. In addition, IL-1β and IL-23 blockade also induced a diminution in the frequency of IL-19^+^ and IL-24^+^ T cells, indicating a novel role for these cytokines in the induction of IL-19 and IL-24 expressing T cells. Finally, elimination of infection resulted in significantly decreased frequencies of antigen – specific CD4^+^ T cells expressing IL-10, IL-19 and IL-24.

**Conclusions:**

Our findings, therefore, suggest that IL-19 and IL-24 are associated with the regulation of immune responses in active filarial infection and potentially with protection against development of pathology, while IL-26 is predominantly associated with pathology in LF.

## Author Summary

Lymphatic filariasis afflicts over 120 million people worldwide. While the infection is mostly clinically asymptomatic, approximately 40 million people suffer from overt, morbid clinical pathology, characterized by swelling of the scrotal area and lower limbs (hydrocele and lymphedema). Host immunologic factors that influence the pathogenesis of disease in these individuals are not completely understood. CD4^+^ and CD8^+^ T cells are known to play a role in promoting pathogenesis through the secretion of pro-inflammatory cytokines, while IL-10 is known to play an important role in dampening inflammation. IL-10 belongs to a family of cytokines that include IL-19, IL-24 and IL-26, known as the IL-10 superfamily. We investigated whether these cytokines have a function similar to IL-10 in individuals with asymptomatic infection and no clinical pathology and those with overt, clinical pathology. We first identify that CD4^+^ and CD8^+^ T cells produce these cytokines. We next identify a significant association of IL-19 and IL-24 secreting T cells with asymptomatic infection. IL-26 secreting T cells, in contrast, appear to be significantly associated with the presence of lymphatic pathology in filarial infection. Therefore, we have uncovered a potentially new regulatory pathway involving the IL-10 superfamily cytokines in filarial infections.

## Introduction

Lymphatic filariasis (LF) is associated with a variety of clinical outcomes [1]. From an immunological perspective, the most intriguing and common clinical manifestation of LF is the subclinical condition associated with circulating microfilariae or circulating adult worm antigen [2]. Clinically asymptomatic LF has been shown to be associated with impaired parasite – specific proliferative responses as well as a down regulation of CD4^+^ T cell responses [3]. While parasite antigens specifically down modulate CD4^+^ Th1 responses, live parasites appear to induce a global down regulation of both Th1 and Th2 responses in vitro [4]. In addition, while the majority of the 120 million infected individuals have subclinical infections, a significant minority (∼40 million) are known to develop lymphatic pathology following infection [5]. The most common pathological manifestations of LF are lymphedema, hydrocele and elephantiasis [5]. Unlike the clinically asymptomatic infection, filarial disease has been associated with increased frequencies of CD4^+^ T cells expressing IFNγ in response to parasite antigen [6] and elevated production of pro-inflammatory Th1 and Th17 cytokines [7] possibly driven by microbial products translocated through lymphatic endothelium [8]. Interestingly, at steady state, patent LF has been shown to be associated with an expanded number of regulatory T cells expressing IL-10 in comparison to uninfected individuals from the same endemic area [9].

Chronic filarial infection has been shown to be associated with a regulatory environment dominated by parasite antigen-driven IL-10 that primarily modulates antigen specific CD4^+^ T cells responses but also provides some spillover suppression of bystander responses as well [10], [11]. While IL-10 has remained the main focus of studies examining immune-regulation in LF and other infectious diseases, it is also known that IL-10 belongs to a superfamily of cytokines that consist of 9 members – IL-10, IL-19, IL-20, IL-22, IL-24, IL-26 and the more distantly related IL-28A, IL-28B and IL-29 [12], [13]. This IL-10 superfamily is felt to be essential for maintaining the integrity and homeostasis of tissues, modulating innate immune responses from tissues to limit the damage caused by viral and bacterial infections and facilitating wound healing processes in infection and inflammation [12], [13]. Unlike IL-10, the role of IL-19, IL-24 and IL-26 in regulating functions of immune cells populations has not been explored. IL-19 has been demonstrated to increase Th2 cytokine expression in activated T cells and activated monocytes and its expression has been shown to be increased in certain Th2-mediated diseases such as atopic dermatitis and asthma [14], [15], [16]. IL-24, in contrast, has been implicated to play an important role in host defense against tumors [17]. More recently, IL-19 and IL-24 have been shown to have an unequivocal immunosuppressive role in the skin by inhibiting the production of IL-1β and IL-17 and promoting infection with *Staphyloccus aureus* [18], whereas IL-26 has been shown to be more pro-inflammatory, as it can induce cytokine production and Th17 cell generation in rheumatoid arthritis [19].

To examine the association of IL-19, IL-24 and IL-26 with immune responses in human filarial infections, we examined the expression pattern of these cytokines in CD4^+^ and CD8^+^ T cells at homeostasis (ex vivo) and following antigen – stimulation in clinically asymptomatic filarial-infected and -uninfected individuals as well as those with disease associated with LF (lymphedema and/or elephantiasis). We demonstrate that the expression pattern of IL-19 and IL-24 in T cells closely mirrors that of IL-10 and that, similar to IL-10, active filarial infection is characterized by elevated frequencies of CD4^+^ and CD8^+^ T cells expressing IL-19 and IL-24. Increased IL-26 expression, in contrast, appears to be associated with the pathological consequences of infection (e.g. lymphedema and/or elephantiasis). In addition, we also demonstrate that this filarial-associated expansion of IL-19 and IL-24 expressing T cells is reversed following successful anti-filarial therapy and that IL-10 itself appears to act as an important regulator of both IL-19 and IL-24 expression. Finally, we also uncover a novel role for the pro-inflammatory cytokines - IL-1β and IL-23, as drivers of the expansion of IL-19 and IL-24 expressing T cells.

## Materials and Methods

### Ethics statement

All individuals were examined as part of natural history studies approved by Institutional Review Boards of both the National Institutes of Allergy and Infectious Diseases and the National Institute for Research in Tuberculosis (NCT00375583 and NCT00001230), and informed written consent was obtained from all participants.

### Study population

We studied a group of 58 clinically asymptomatic infected (hereafter INF) individuals, 23 individuals with filarial lymphedema (hereafter CP) individuals and 15 uninfected, endemic normal (hereafter UN) individuals in a *W. bancrofti*-area endemic Tamil Nadu, South India (Table 1). All CP and UN individuals were circulating filarial antigen negative by both the ICT filarial antigen test (Binax, Portland, ME) and the TropBio Og4C3 enzyme-linked immunosorbent assay (ELISA) (Trop Bio Pty. Ltd, Townsville, Queensland, Australia) indicating a lack of current active infection. The diagnosis of prior filarial infection in those with CP was made by history and clinical examination as well as positive *Brugia malayi* antigen (BmA) -specific IgG4. BmA-specific IgG4 and IgG ELISA were performed exactly as described previously [20]. All INF individuals tested positive for by both the ICT filarial antigen test and the TropBio Og4C3 ELISA and had not received any anti-filarial treatment prior to this study. The UN individuals were from the same area/community as the INF individuals and were recruited during the same time period. All INF individuals were treated with a standard dose of diethylcarbamazine and albendazole and follow – up blood draws were obtained one year later. A subset of individuals from the initial set of INF individuals who became circulating antigen negative (Cured, n = 9) and another subset who remained antigen positive (Not cured, n = 7) following treatment were used for post-treatment analysis. Another set of 17 INF individuals were used for the in vitro cytokine blocking and addition studies. There were no differences between the groups in terms of demographics or socio-economic status.

**Table 1:** Characteristics of the study population.

	CP^a^ (n = 23)	INF (n = 58)	UN (n = 15)
Median age (range)	42 (27–65)	39 (19–65)	36 (24–65)
Gender male/female	17/6	38/20	9/6
Lymphedema/Elephantiasis	Yes	None	None
ICT card test	Negative	Positive	Negative
*W. bancrofti* circulating antigen levels (U/ml) [GM(Range)]	<32^b^	3808 (138–25680	<32 (138–25680)

a CP refers to individuals with filarial pathology, INF refers to individuals with asymptomatic, filarial infection and UN refers to uninfected individuals.

b Below the limits of detection.

### Parasite and control antigen

Saline extracts of *B. malayi* adult worms (BmA) and microfilariae (Mf) were used for parasite antigens and mycobacterial PPD (Serum Statens Institute, Copenhagen, Denmark) was used as a control antigen. Final concentrations were 10 µg/ml for BmA, Mf and PPD. Endotoxin levels in the BmA was <0.1 EU/mg using the QCL-1000 Chromogenic LAL test kit (BioWhittaker). Phorbol myristoyl acetate (PMA) and ionomycin at concentrations of 12.5 ng/ml and 125 ng/ml (respectively) were used as the positive control stimuli.

### Isolation of PBMC and culture

Heparinized blood was collected and PBMC isolated by Ficoll diatrizoate gradient centrifugation (LSM; ICN Biomedicals). Erythrocytes were lysed using ACK lysis buffer (Biosource). Cells were then washed and cultured in RPMI-1640 (BioWhittaker), supplemented with 20 mM glutamine (BioWhittaker), 10% heat-inactivated FCS (Harlan Bioproducts for Science), and 50 µg/ml of gentamicin (Mediatech). PBMC were cultured with BmA, Mf, PPD or PMA/ionomcyin in 24-well tissue culture plates (Corning) at concentrations of 5×10^6^/well. After 24 h, culture supernatants were collected and analyzed for cytokines.

### In vitro whole blood culture

Whole blood cell cultures were performed to determine the intracellular levels of cytokines. Briefly, whole blood was diluted 1∶1 with RPMI-1640 medium, supplemented with penicillin/streptomycin (100 U/100 mg/ml), L-glutamine (2 mM), and HEPES (10 mM) (all from Invitrogen, San Diego, CA) and placed in 12-well tissue culture plates (Costar, Corning Inc., NY, USA). The cultures were then stimulated with BmA, Mf, PPD, PMA/ionomycin (P/I) or media alone in the presence of a costimulatory reagent CD49d/CD28 (BD Biosciences) at 37°C for 6 hrs. FastImmune Brefeldin A Solution (10 µg/ml) (BD Biosciences) was added after 2 hours. After 6 hours, the whole blood was centrifuged, washed using cold PBS, and then 1× FACS lysing solution (BD Biosciences, San Diego, CA, USA) was added. The cells were fixed using cytofix/cytoperm buffer (BD Biosciences, San Diego, CA, USA), cryopreserved by the addition of 1∶10 PBS/DMSO and stored at −80°C until used. For cytokine neutralization experiments, whole blood from INF individuals (n = 10) was cultured in the presence of anti-IL-10 (5 µg/ml), anti-IL-1β (5 µg/ml), anti-IL-23R (5 µg/ml) or isotype control antibody (5 µg/ml) (R& D Sytems) for 1 h following which BmA and brefeldin A was added and cultured for a further 23 h. For IL-10 addition experiments, whole blood from INF individuals (n = 7) was cultured with BmA and 20 ng/ml of recombinant IL-10 (rIL-10, R&D Systems) for 24 h.

### Intracellular cytokine staining

The cells were thawed and washed with PBS first and PBS/1% BSA later and then stained with surface antibodies for 30–60 minutes. Surface antibodies used were CD3 - Amcyan, CD4 - APC-H7 and CD8 - PE-Cy7 (all from BD Biosciences). The cells were washed and permeabilized with BD Perm/Wash buffer (BD Biosciences) and stained with intracellular cytokines for an additional 30 min before washing and acquisition. Cytokine antibodies used were IL-10 (BD Pharmingen), IL-19, IL-24 and IL-26 (R&D Systems). Flow cytometry was performed on a FACS Canto II flow cytometer with FACSDiva software v.6 (Becton Dickinson). The lymphocyte gating was set by forward and side scatter and 100,000 lymphocyte events were acquired. FMO gating was used for intracellular cytokine detection. Data were collected and analyzed using Flow Jo software. All data are depicted as frequency of CD4^+^ or CD8^+^ T cells expressing cytokine(s). Values following media stimulation are depicted as baseline frequency while frequencies following stimulation with antigens or anti-CD3 are depicted as net frequencies (with baseline values subtracted).

### ELISA

The levels of IL-19, IL-24 and IL-26 in the PBMC culture supernatants were measured using the kit from R&D Systems.

### RNA preparation

RNA was isolated from PBMCs following culture with BmA or rIL-10 for 24 h. PBMCs were lysed using the reagents of a commercial kit (QIAshredder; Qiagen, Valencia, CA). Total RNA was extracted according to the manufacturer's protocol (RNeasy mini kit; Qiagen), and RNA was dissolved in 50 ml of RNase-free water.

### cDNA synthesis

RNA (1 µg) was used to generate cDNA using TaqMan reverse transcription reagents according to the manufacturer's protocol (Applied Biosystems, Fullerton, CA). Briefly, random hexamers were used to prime RNA samples for reverse transcription using MultiScribe reverse transcriptase.

### Real-time RT-PCR

Real-time quantitative RT-PCR was performed in an ABI 7500 sequence detection system (Applied Biosystems) using TaqMan Assays-on-Demand reagents for IL-19, IL-24, IL-26 and an endogenous 18s ribosomal RNA control. Relative transcript levels were determined according to the manufacturer's protocol.

### Statistical analysis

Data analyses were performed using GraphPad PRISM (GraphPad Software, Inc., San Diego, CA, USA). Geometric means (GM) were used for measurements of central tendency. Comparisons were made using either the Kruskal-Wallis test with Dunn's multiple comparisons or Mann-Whitney U test (unpaired comparisons) or the Wilcoxon signed rank test (paired comparisons). Multiple comparisons were corrected using the Holm's correction.

## Results

### Filarial infection is associated with increased frequencies of spontaneous and antigen–specific CD4^+^ T cells expressing IL-19 and IL-24

**Figure 1:**
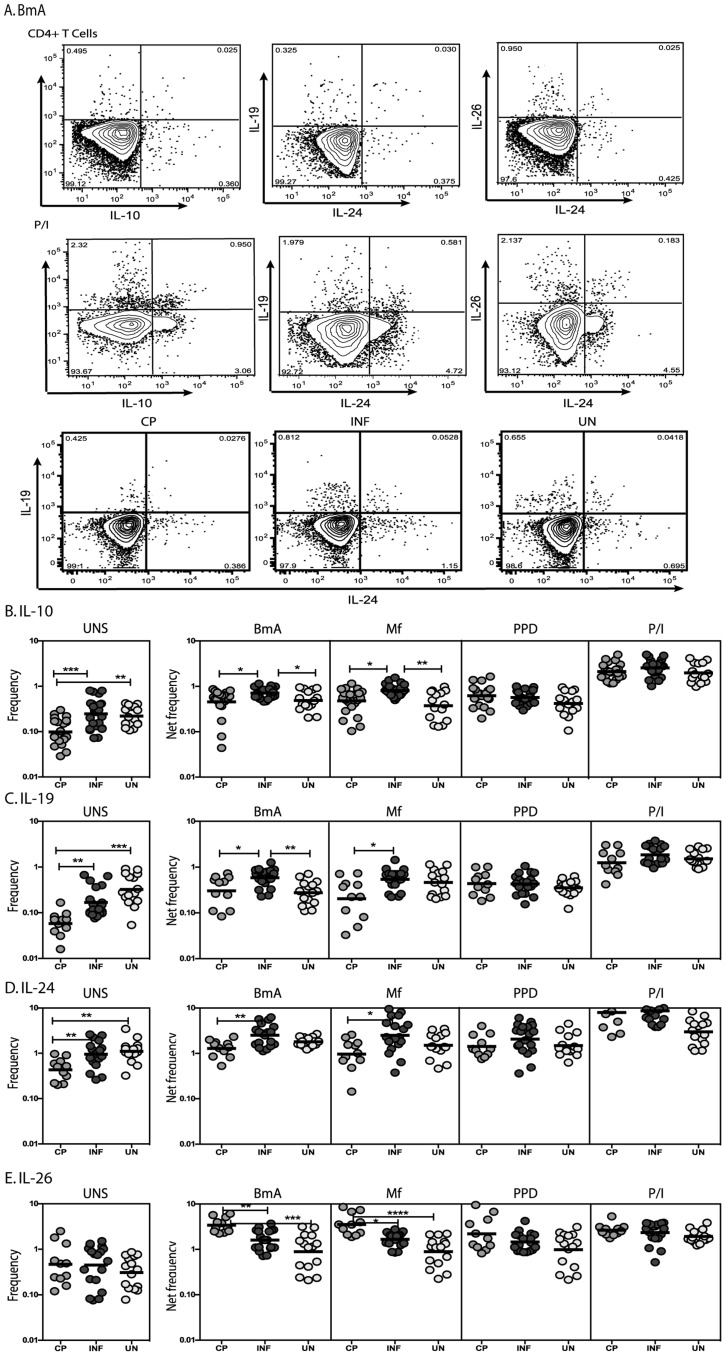
Filarial infection is associated with increased frequencies of IL-10, IL-19 and IL-24 expressing and decreased frequencies of IL-26 expressing CD4^+^ T cells. (A) A representative dot plot from a filarial - infected individual showing CD4^+^ T cell expression of IL-10, IL-19, IL-24 and IL-26 in response to BmA and PMA/Ionomycin and representative dot plots from CP, INF and UN individuals for CD4^+^ T cell expression of IL-19 and IL-24. The frequencies of CD4^+^ T cells expressing IL-10 (B), IL-19 (C), IL-24 (D) and IL-26 (E) at baseline and following stimulation with BmA, Mf, PPD and PMA/ionomycin in CP (n = 23), INF (n = 25) and UN (n = 15) individuals. Antigen – stimulated frequencies are shown as net frequencies with the baseline levels subtracted. The data are shown as scatter plots with each circle representing a single individual. *P* values were calculated using the Kruskal-Wallis test with Dunn's multiple comparisons (* p<0.05, ** p<0.01, *** p<0.001).

To determine the association of IL-10 and the related family members IL-19, IL-24 and IL-26 in protection from pathology and in susceptibility to or resistance to clinically apparent disease in LF, we measured the frequency of CD4^+^ T cells expressing these cytokines in INF individuals and in those with LF-associated disease (CP) or UN individuals (Figure 1A). As shown in Figure 1B, INF individuals had significantly increased frequencies of CD4^+^ T cells expressing IL-10 at homeostasis/steady state in comparison to CP individuals and increased frequencies of CD4^+^ T cells expressing IL-10 upon filarial antigen stimulation compared to CP and UN individuals. Similarly, INF individuals also exhibited significantly increased frequencies of CD4^+^ T cells expressing IL-19 at baseline in comparison to CP individuals and following filarial antigen stimulation compared to CP and UN individuals (Figure 1C). In addition, INF had significantly increased frequencies of CD4^+^ T cells expressing IL-24 at baseline and following filarial antigen stimulation in comparison to CP individuals (Figure 1D). In contrast, INF had significantly lower frequencies of CD4^+^ T cells expressing IL-26 following filarial antigen stimulation compared to CP individuals (Figure 1E). Interestingly, no significant difference was observed in the frequency of CD4^+^ T cells expressing IL-10, IL-19, IL-24 or IL-26 upon PPD stimulation or following PMA/ionomycin stimulation. Thus, filarial infection is associated with elevated frequencies of spontaneous and/or antigen – specific IL-10, IL-19 and IL-24 expressing CD4^+^ T cells in comparison to CP individuals, suggesting that these cells are associated with protection against pathology. In addition, filarial infection is also associated with decreased frequencies of antigen – specific IL-26 expressing CD4^+^ T cells in comparison to CP individuals, suggesting that IL-26 expressing T cells are pre-dominantly associated with pathology.

### Filarial infection is associated with increased frequencies of antigen – specific CD8^+^ T cells expressing IL-19 and decreased frequencies of CD8^+^ T cells expressing IL-26

**Figure 2:**
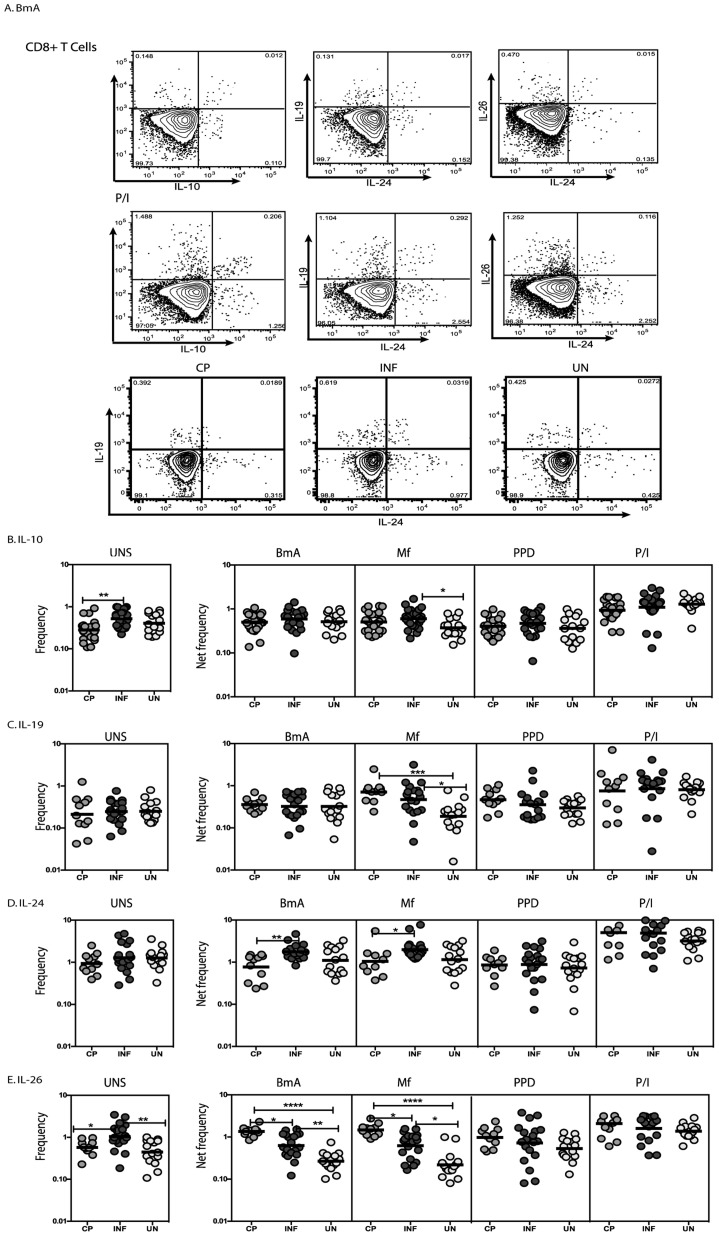
Filarial infection is associated with increased frequencies of IL-24 expressing and decreased frequencies of IL-26 expressing CD8^+^ T cells. (A) A representative dot plot from a filarial - infected individual showing CD8^+^ T cell expression of IL-10, IL-19, IL-24 and IL-26 in response to BmA and PMA/Ionomycin and representative dot plots from CP, INF and UN individuals for CD4^+^ T cell expression of IL-19 and IL-24. The frequencies of CD8^+^ T cells expressing IL-10 (B), IL-19 (C), IL-24 (D) and IL-26 (E) at baseline and following stimulation with BmA, Mf, PPD and PMA/ionomycin in CP (n = 23), INF (n = 25) and UN (n = 15) individuals. Antigen – stimulated frequencies are shown as net frequencies with the baseline levels subtracted. The data are shown as scatter plots with each circle representing a single individual. *P* values were calculated using the Kruskal-Wallis test with Dunn's multiple comparisons (* p<0.05, ** p<0.01, *** p<0.001).

To determine the association of CD8^+^ T cell expression of IL-10 and its related family members IL-19, IL-24 and IL-26 in LF, we measured the frequency of CD8^+^ T cells expressing these cytokines in INF individuals and in those with LF-associated disease (CP) and UN individuals (Figure 2A). As shown in Figure 2B, INF individuals had significantly higher spontaneous frequencies of CD8^+^ T cells expressing IL-10 at baseline but no alteration following filarial antigen - stimulation in INF compared to CP individuals. Similarly, as shown in Figure 2C, INF individuals had no significant alteration in the baseline or filarial - antigen stimulated frequency of CD8^+^ T cells expressing IL-19 compared to UN individuals. However, INF individuals exhibited significantly increased frequencies of CD8^+^ T cells expressing IL-24 following filarial antigen stimulation compared to CP individuals (Figure 2D). In contrast, INF had significantly lower frequencies of CD8^+^ T cells expressing IL-26 following filarial antigen stimulation compared to CP individuals (Figure 2E). Similar to CD4^+^ T cells, no significant difference was observed in the frequency of CD8^+^ T cells expressing IL-10, IL-19, IL-24 or IL-26 upon PPD stimulation or following PMA/ionomycin stimulation. Thus, filarial infection is associated with elevated frequencies of antigen – specific IL-24^+^ and diminished frequencies of IL-26^+^ CD8^+^ T cells in comparison to individuals with chronic pathology, suggesting that these cells are also associated with the presence of pathology.

### Filarial infection is associated with increased production of antigen – specific IL-19 and IL-24 and decreased production of IL-26

**Figure 3:**
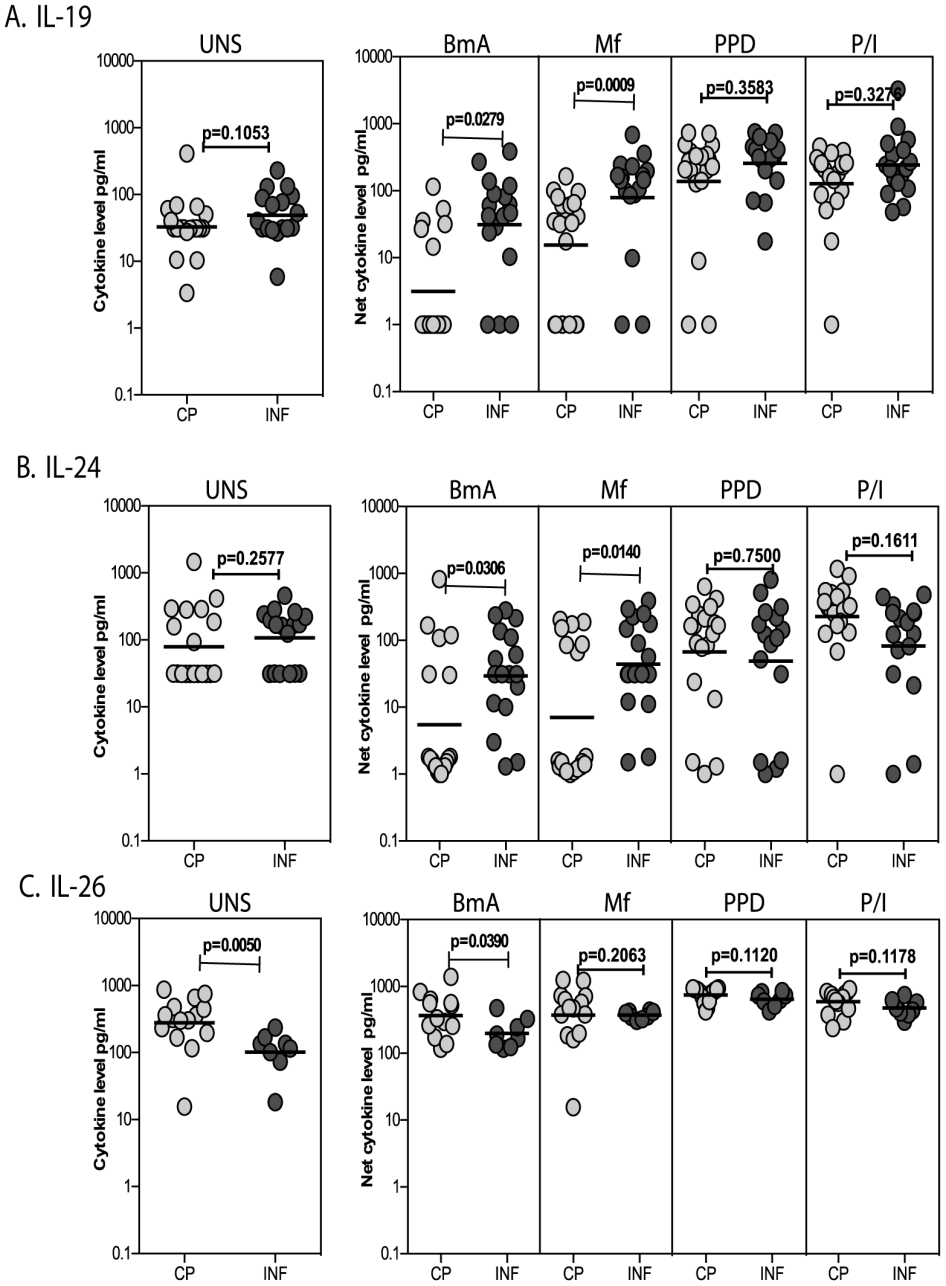
Filarial infection is associated with increased levels of antigen - induced IL-19 and IL-24 and decreased levels of IL-26. The total levels of IL-19 (A), IL-24 (B) and IL-26 (C) in PBMC culture supernatants at baseline or following stimulation with BmA, Mf, PPD or PMA/ionomycin in CP (n = 19) and INF (n = 17) individuals. Antigen – stimulated cytokine levels are shown as net cytokine levels with baseline subtracted. The data are shown as scatter plots with each circle representing a single individual or as bar graphs with geometric means and 95% confidence intervals. P values were calculated using the Mann-Whitney test.

To determine whether the T cell expression pattern of IL-19, IL-24 and IL-26 is reflected in secreted cytokine production following antigen stimulation, we cultured PBMC from INF and CP individuals with filarial and non-filarial antigen (PPD) as well as with PMA/ionomycin as a positive stimulus for 24 h and measured the levels of IL-19, IL-24 and IL-26 by ELISA. As shown in Figure 3A, both BmA [Geometric Mean (GM) of 31.3 pg/ml versus 3.1 pg/ml] and Mf (GM of 99.4 vs. 15.6) induced significantly higher production of IL-19 in PBMC from INF compared to CP individuals. As also shown in Figure 3B, both BmA (GM of 29.4 vs. 5.5) and Mf (GM of 44.2 vs. 7.1) induced significantly higher production of IL-24 in PBMC from INF compared to CP individuals. In contrast, as shown in Figure 3C, BmA induced significantly lower production of IL-26 from INF compared to CP individuals (GM of 368 vs. 198). This response was filarial – antigen-specific since neither PPD nor PMA/ionomycin induced any significant differences in the net cytokine production of IL-19, IL-24 or IL-26. Similarly, spontaneous production of IL-19 and IL-24 (but not IL-26) was also not significantly different between INF and CP individuals. Thus, filarial infection is characterized by increased production of parasite antigen – specific IL-19 and IL-24 and decreased production of IL-26.

### IL-10 as well as IL-1β and IL-23 regulate the expression of IL-19 and IL-24 in CD4^+^ and CD8^+^ T cells

**Figure 4:**
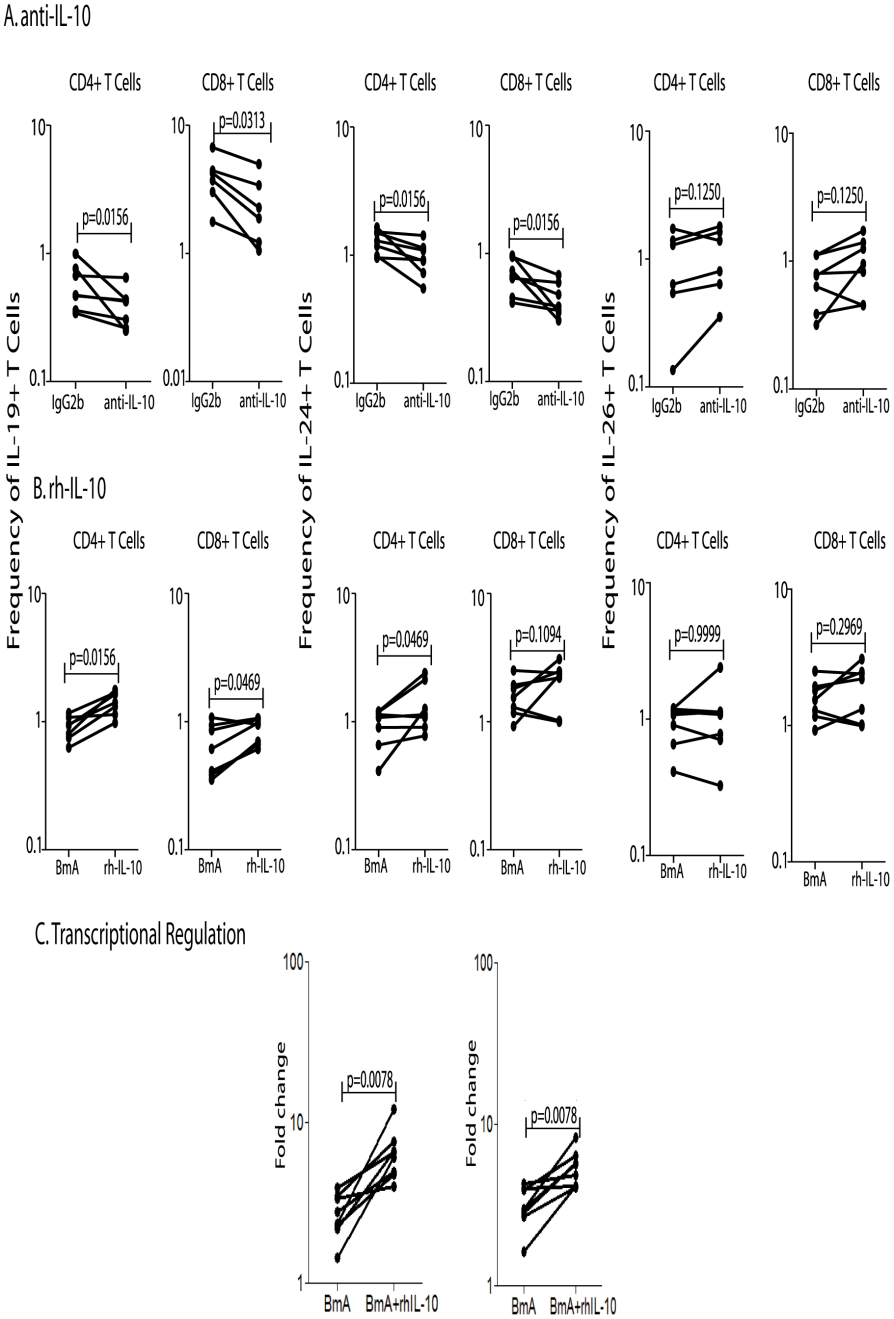
IL-10 regulates the frequencies of CD4^+^ and CD8^+^ T cells expressing IL-19 and IL-24 and modulates IL-19 and IL-24 gene expression in filarial infections. (A) The frequencies of CD4^+^ and CD8^+^ T cells expressing IL-19, IL-24 and IL-26 following IL-10 neutralization and stimulation with BmA in a subset of INF individuals (n = 7). (B) The frequencies of CD4^+^ and CD8^+^ T cells expressing IL-19, IL-24 and IL-26 following addition of rhIL-10 and stimulation with BmA in a subset of INF individuals (n = 7). Antigen – stimulated frequencies are shown as net frequencies with the baseline levels subtracted. (C) PBMCs from infected (INF) individuals (*n* = 7) were cultured with BmA alone or BmA with rhIL-10 for 24 h and mRNA expression of IL-19, IL-24 and 18s RNA were measured by RT-PCR. Data are shown as fold change BmA plus IL-10 over BmA alone controls. Each line represents a single individual. P values were calculated using the Wilcoxon signed rank test.

To determine the role of IL-10 in regulating the expression patterns of CD4^+^ and CD8^+^ T cells expressing IL-19, IL-24 and IL-26 in filarial infection, we measured the frequency of CD4^+^ and CD8^+^ T cells expressing these cytokines in the presence of an anti-IL-10 neutralizing antibody or of an isotype control in a subset of INF individuals (n = 7); we also examined the addition of recombinant IL-10 in a separate subset of INF individuals (n = 7). As shown in Figure 4A, IL-10 neutralization resulted in significantly decreased frequencies of CD4^+^ and CD8^+^ T cells expressing IL-19 and IL-24 in INF individuals. Conversely, the addition of rIL-10 to parasite-stimulated whole blood cultures from INF individuals resulted in a significant increase in the frequency of CD4^+^ and CD8^+^ T cells expressing IL-19 and IL-24 (Figure 4B). However, neither anti-IL-10 nor rIL-10 had any significant effect on IL-26^+^ T cells in INF individuals (Figure 4). Interestingly, we observed a similar effect on UN individuals as well (data not shown).

Since, IL-10 altered the frequency of cytokine expressing T cells, we sought to determine if IL-10 altered the transcriptional regulation of IL-19 and IL-24. To this end, we examined the gene expression of IL-19 and IL-24 in RNA from PBMC of INF individuals (n = 7) following addition of rIL-10 and BmA. As shown in Figure 4C, rIL-10 induced significant upregulation of the transcripts for IL-19 (GM fold change of 3.8) and IL-24 (GM fold change of 4.2) compared to the media control following BmA stimulation. Thus, both IL-19 and IL-24 expression appears to be regulated at the transcriptional level by IL-10 in filarial infections.

**Figure 5:**
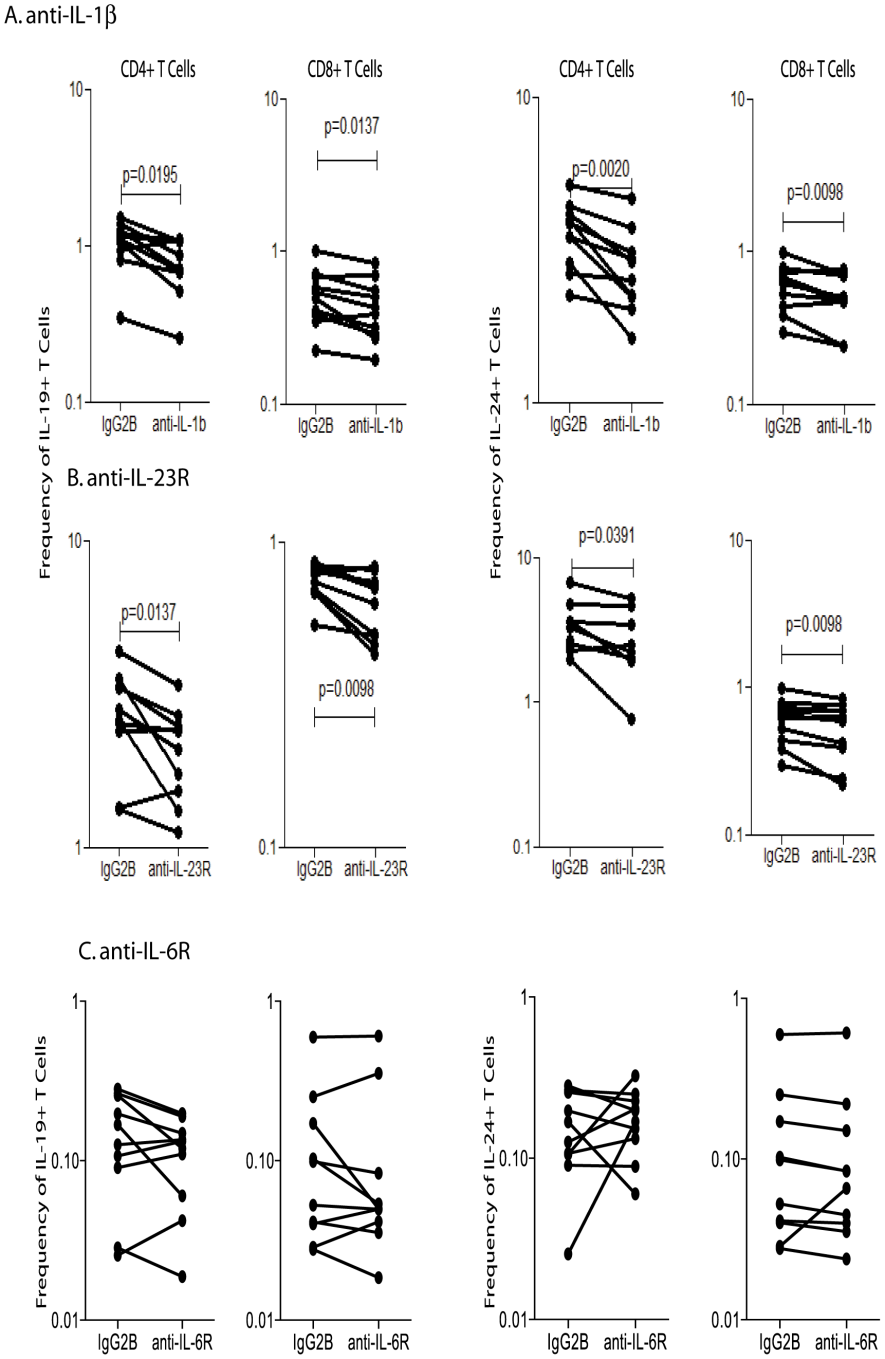
IL-1β and IL-23 regulate the frequencies of CD4^+^ and CD8^+^ T cells expressing IL-19 and IL-24 in filarial infections. (A) The frequencies of CD4^+^ and CD8^+^ T cells expressing IL-19 and IL-24 following IL-1β neutralization and stimulation with BmA in a subset of INF individuals (n = 10). (B) The frequencies of CD4^+^ and CD8^+^ T cells expressing IL-19 and IL-24 following blockade of IL-23R and stimulation with BmA in a subset of INF individuals (n = 10). (C) The frequencies of CD4^+^ and CD8^+^ T cells expressing IL-19 and IL-24 following blockade of IL-6R and stimulation with BmA in a subset of INF individuals (n = 10). Antigen – stimulated frequencies are shown as net frequencies with the baseline levels subtracted. Each line represents a single individual. P values were calculated using the Wilcoxon signed rank test.

We also tested the role of other pro- and anti-inflammatory cytokines in inducing or abrogating the expansion of CD4^+^ and CD8^+^ T cells expressing IL-10 family cytokines. Among the cytokines tested, (IL-1β, IL-2, IL-4, IL-6, IL-23 and TGFβ), only IL-1β and IL-23 exhibited any significant effect on the induction of IL-19 and IL-24 expressing T cells. As shown in Figure 5A, blockade of IL-1β resulted in significantly lower frequencies of CD4^+^ and CD8^+^ T cells expressing IL-19 and IL-24 in INF individuals (n = 10). Similarly, blockade of IL-23R also resulted in significantly lower frequencies of CD4^+^ and CD8^+^ T cells expressing IL-19 and IL-24 (Figure 5B). On the other hand, blockade of IL-6R had no significant effect on the frequencies of CD4^+^ and CD8^+^ T cells expressing IL-19 and IL-24 (Figure 5C). None of these cytokines had any significant effect on the frequencies of IL-26 expressing T cells in filarial infection (data not shown). Thus, IL-1β and IL-23 appear to also regulate IL-19 and IL-24 expression in T cells.

### Treatment of filarial infection alters the frequencies of antigen – specific CD4^+^ and CD8^+^ T cells expressing IL-10, IL-19, IL-24 and IL-26

**Figure 6:**
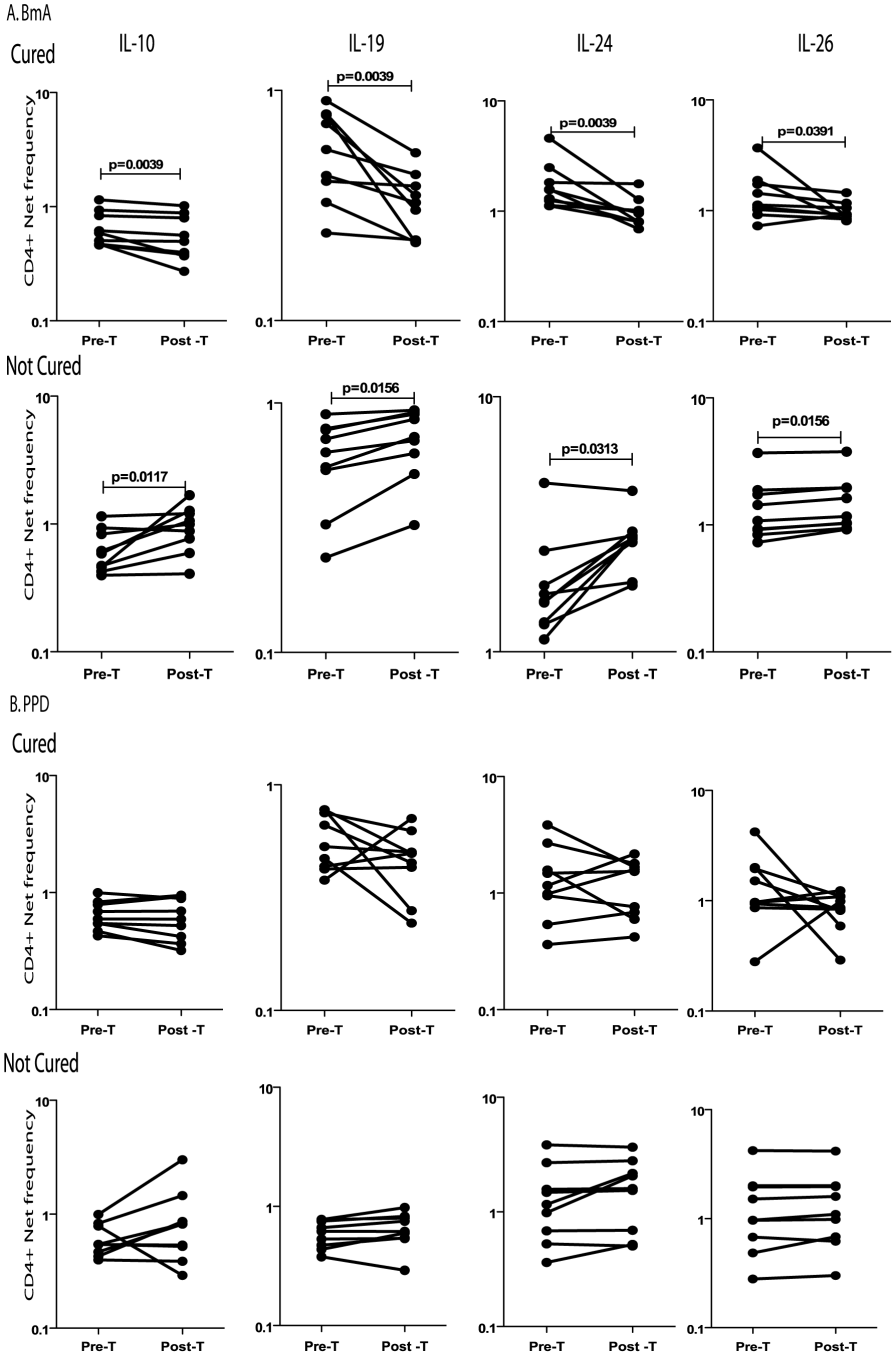
Treatment of filarial infection is associated with alterations in the frequency of CD4^+^ T cells expressing IL-10, IL-19, IL-24 and IL-26. The frequencies of CD4^+^ T cells expressing IL-10, IL-19, IL-24 and IL-26 following stimulation with BmA (A) or PPD (B) before and after treatment with a standard dose of DEC and albendazole in a subset of INF individuals (Cured, n = 9), who turned circulating antigen negative and another set of INF individuals (Not cured, n = 7), who remained circulating antigen positive. Antigen – stimulated frequencies are shown as net frequencies with the baseline levels subtracted. Each line represents a single individual. P values were calculated using the Wilcoxon signed rank test.

To determine the role of antigen – persistence in the maintenance of CD4^+^ and CD8^+^ T cells expressing IL-19 and IL-24 in filarial infections, we measured the frequency of CD4^+^ and CD8^+^ T cells expressing these IL-19, IL-24, and IL-10 in two subsets of INF individuals, one subset having eliminated infection as demonstrated by the absence of circulating filarial antigen (Cured, n = 9) and the other who continued to harbor infection (Not cured, n = 7) following administration of anti-filarial chemotherapy. As shown in Figure 6A, treatment of filarial infection and consequent cure resulted in significantly decreased frequencies of CD4^+^ T cells expressing IL-10, IL-19, IL-24 and IL-26 after parasite-antigen stimulation whereas those who continued to harbor infection did not exhibit reduction in these frequencies. In contrast, PPD stimulation did not have any significant effect on the frequencies of CD4^+^ T cells expressing IL-10, IL-19, IL-24 and IL-26 (Figure 6B). These data together indicate that effect of anti-filarial treatment was parasite-specific. This was further confirmed by the finding that while Mf antigen also inhibited the frequencies of CD4^+^ T cells expressing the above cytokines in cured but not in uncured individuals (Figure S1A), PMA/ionomycin did not exhibit any differential effect on the cytokine expressing CD4^+^ T cell frequencies (Figure S1B). In addition, we observed a very similar effect on the frequencies of CD8^+^ T cells upon stimulation with filarial antigens or PPD or PMA/ionomcyin in treated individuals with or without cure (Figure S2). Thus, the regulation of IL-10, IL-19 and IL-24 expression in filarial infection is dependent on the presence of active infection and therefore, curative treatment ablates the increase in the frequency of these predominantly regulatory T cells.

## Discussion

LF is characterized by a diverse set of clinical manifestations including an asymptomatic (or subclinical) form seen among the majority of infected people [2]. Filarial parasites exert profound immunoregulatory effects on the host immune system with both parasite-antigen specific and more generalized levels of immune suppression [11]. Among the host factors influencing immune-regulation, one of the key players is IL-10. IL-10 is a powerful immune-regulatory cytokine known to be induced in a variety of helminth infections [21]. The IL-10 dominated regulatory environment induced in chronic helminth infections is known to modulate the entire repertoire of CD4^+^ and CD8^+^ T cell effector functions [21]. Increased levels of spontaneous as well as parasite – specific IL-10 are associated with filarial infections [22] and thought to play a crucial role in down-regulation of T cell-mediated immune responses [21]. Therefore, this IL-10 dominated response has the potential to regulate not only the balance of T cell subsets, but also to modulate the response to both bystander antigens and allergens as well [11], [23], [24]. Less well studied, however, is the role of other cytokines belonging to the IL-10 superfamily that includes IL-19, IL-20, IL-22, IL-24 and IL-26 [12]. These cytokines elicit diverse host defense responses during infections and are known to facilitate the tissue-healing process in infection and inflammation [12]. With the exception of IL-22 (whose role as a Th17 cytokine has been well characterized) [25], the role of these IL-10 family of cytokines in modulating immune responses in chronic infection is not known. Although IL-19 has been shown to be increased in both Th1- and Th2-dominant diseases such as psoriasis and asthma, respectively [15], [16], data from IL-19 deficient mice and human data autoimmune diseases and other inflammatory conditions suggest that IL-19 has a potent anti-inflammatory activity [26], [27], [28]. IL-24, alternatively, has been predominantly studied in tumor immunology and has exhibited great promise as an anti-tumor therapeutic cytokine [17]. Recent data from a mouse model of *S. aureus*, however, clearly reveals an important immuno-modulatory role for IL-19 and IL-24 in suppressing IL-1β and IL-17 dependent effector pathways and promoting susceptibility to infection [18].

Since CD4^+^ T cells are known producers of IL-19 and IL-24 [12], we studied the regulation of these cytokines as well as IL-26 in filarial infection. Our study of CD4^+^ and CD8^+^ T cells expressing IL-10 and its extended family reveals four important features. First, CD4^+^ T cells expressing IL-19 and IL-24 mirror the pattern observed in CD4^+^ T cells expressing IL-10. Thus, both spontaneously and/or following filarial – antigen stimulation, we consistently detected a significantly higher proportion of CD4^+^ T cells expressing IL-10, IL-19 and IL-24 in the asymptomatic, infected group. Interestingly, in our study, the spontaneous frequency of IL-10 expressing CD4^+^ T cells was similar between the INF and UN groups. Whether this is attributable to non-specific potentiation of IL-10 production in T cells in these individuals is unclear at this point. Second, CD4^+^ and CD8^+^ T cells expressing IL-26 show a very different pattern of expression with significantly decreased frequencies being observed following antigen – stimulation suggesting that IL-26^+^ T cells are associated with inflammatory pathology rather than the asymptomatic state. Third, the regulation of IL-10, IL-19, IL-24 and IL-26 in T cells appears to be highly antigen – specific since the elevated frequencies of IL-10, IL-19, IL-24 and IL-26 expressing T cells is observed only upon filarial antigen stimulation but not following stimulation with an unrelated antigen (PPD) or with a mitogen. Finally, the filarial antigen induced increase in IL-10^+^ or IL-19^+^ T cells in INF individuals or IL-26^+^ T cells in CP individuals is significant even when compared to UN individuals, indicating that the alterations in expression pattern of IL-10 associated cytokines is directly associated with filarial infection or disease. Our report, while not providing direct mechanistic evidence, nevertheless suggests a putative role for the IL-10 family of cytokines especially IL-19 and IL-24 in mediating protection against pathology, as does IL-10 itself. This is further corroborated by the fact that filarial - antigen stimulation of PBMC cultures also resulted in significantly increased levels of total IL-19 and IL-24 in filarial infected individuals when compared to those with pathology and inactive infection. Our data also reveal an important association of IL-26 expressing T cells with filarial lymphedema. This association is similar to the role that IL-26 plays in other inflammatory diseases such as rheumatoid arthritis [19] – wherein IL-26 has been shown to be over-expressed and to generate pro-inflammatory cytokine production and Th17 cell induction – and Crohn's disease [29] – wherein IL-26 modulates intestinal epithelial cell proliferation and pro-inflammatory gene expression. Although we have not explored the mechanism by which IL-26 regulates or exacerbates pathology in LF, the presence of elevated frequencies of IL-26 expressing T cells in filarial lymphedema clearly suggests an association with pathology. Finally, a possible explanation for differential relationship between the effector functions of IL-19/IL-24 and those of IL-26 may be related to receptor usage among these cytokines. While IL-19 and IL-24 signal predominantly through the Type 1 IL-20 receptor (composed of IL-20R1 and IL-20R2), IL-26 signaling occurs through a hetero-dimeric receptor composed of IL-10R2 and IL-20R1 [12].

In addition to characterizing the expression pattern, we also examined some of the mechanisms regulating the expression of these cytokines. Since IL-10 and the persistence of antigen in chronic infections are known to play a role in modulating T cell expression of cytokines [10], we examined the frequencies of CD4^+^ and CD8^+^ T cells expressing IL-19, IL-24 and IL-26 following IL-10 blockade during in vitro stimulation and following addition of rIL-10. Our findings reveal an important role for IL-10 in the induction of IL-19 and IL-24 as IL-10 blockade resulted in partial abrogation of CD4^+^ and CD8^+^ T cells expression of these cytokines. Our data also reveal that the addition of exogenous rIL-10 significantly modulated IL-19 and IL-24 expression by T cells and suggest that IL-10 may be a transcriptional regulator of IL-19 and IL-24 in filarial infections. Moreover, this regulation is also observed in UN individuals (data not shown) indicating that IL-10 regulation of IL-19 and IL-24 is a generic regulatory response. While it would be tempting to speculate that IL-10 induction in filarial infection is mainly responsible for the subsequent induction of IL-19 and IL-24, our data did not examine the kinetics of induction of these cytokines in filarial infection. In terms of other regulators of IL-19 and IL-24, our data suggest that IL-1β and IL-23 induce the expression of IL-19 and IL-24. To our knowledge, this is the first piece of evidence for a role of IL-1β in regulating IL-10 superfamily members, whereas there has been one previous report demonstrating that IL-23 can induce IL-19 and IL-24 in mouse epidermal cells [30]. Interestingly, neither IL-10 itself not IL-1β and IL-23 had any effect in regulating the expression of IL-26 in CD4^+^ and CD8^+^ T cells. Whether this differential regulation reflects differences in proximity (at the chromosomal level) or genomic structures between IL-10 and the other family members [12] awaits clarification.

One of the hallmarks of chronic infection is the modulation of immune responses in the presence of active infection. However, very little is known about the regulation of these immuno-modulatory pathways after the elimination of infection. Indeed, although IL-10 has been thoroughly investigated as the dominant immune modulator in lymphatic filariasis, no data exist on the T cell expression of IL-10 following successful chemotherapy. Therefore, we examined the frequencies of IL-10^+^, IL-19^+^, IL-24^+^ and IL-26^+^ T cells in patients with active LF before and following anti-filarial chemotherapy (in which there was subsequent elimination of filarial antigens). As a control, we also examined individuals who underwent treatment but failed to eliminate infection. Our findings reveal that curative therapy is associated with changes in the cytokine secreting repertoire of CD4^+^ and CD8^+^ T cells. Thus, the frequency of T cells expressing IL-10, IL-19, IL-24 and IL-26 are all decreased upon curative treatment, in contrast to that seen in those who continued to harbor infection. This reduction in frequencies might represent a reduction in the total frequency of filarial - specific T cells. Nevertheless, our study therefore demonstrates that persistence of antigen is a critical factor in maintaining the elevated frequencies of IL-10, IL-19 and IL-24 expressing T cells in filarial infections.

In summary, we have investigated the role of the IL-10 family of cytokines in filarial infection and disease. While we have not performed decades long longitudinal studies to define the development of pathology in filarial infection, our strategy of contrasting immune responses in individuals with subclinical disease and those with chronic clinical manifestations has yielded important information on the role of IL-10, IL-19, IL-24 and IL-26 in pathogenesis. Future studies to evaluate the mechanism by which IL-19 and IL-24 regulate immune response in LF and the mechanism by which IL-26 is associated with pathogenesis should shed light on the role of the IL-10 family of cytokines in other chronic infections as well.

## Supporting Information

Figure S1 **Treatment of filarial infection is associated with alterations in the frequency of CD4^+^ T cells expressing IL-10, IL-19, IL-24 and IL-26.** The frequencies of CD4^+^ T cells expressing IL-10, IL-19, IL-24 and IL-26 following stimulation with Mf (A) or PMA/ionomycin (B) before and after treatment with a standard dose of DEC and albendazole in a subset of INF individuals (Cured, n = 9), who turned circulating antigen negative and another set of INF individuals (Not cured, n = 7), who remained circulating antigen positive. Antigen – stimulated frequencies are shown as net frequencies with the baseline levels subtracted. Each line represents a single individual. P values were calculated using the Wilcoxon signed rank test.
(TIF)

Figure S2 **Treatment of filarial infection is associated with alterations in the frequency of CD8^+^ T cells expressing IL-10, IL-19, IL-24 and IL-26.** The frequencies of CD8^+^ T cells expressing IL-10, IL-19, IL-24 and IL-26 following stimulation with BmA (A), Mf (B), PPD (C) and PMA/ionomycin (D) before and after treatment with a standard dose of DEC and albendazole in a subset of INF individuals (Cured, n = 9), who turned circulating antigen negative and another set of INF individuals (Not cured, n = 7), who remained circulating antigen positive. Antigen – stimulated frequencies are shown as net frequencies with the baseline levels subtracted. Each line represents a single individual. P values were calculated using the Wilcoxon signed rank test.
(TIF)
