# Supplementary figures and images for: Expansion of Parasite-Specific CD4+ and CD8+ T Cells Expressing IL-10 Superfamily Cytokine Members and Their Regulation in Human Lymphatic Filariasis
Source: PLoS Negl Trop Dis. 2014 Apr 3;8(4):e2762. doi: 10.1371/journal.pntd.0002762 (PMC3974669; doi:10.1371/journal.pntd.0002762)

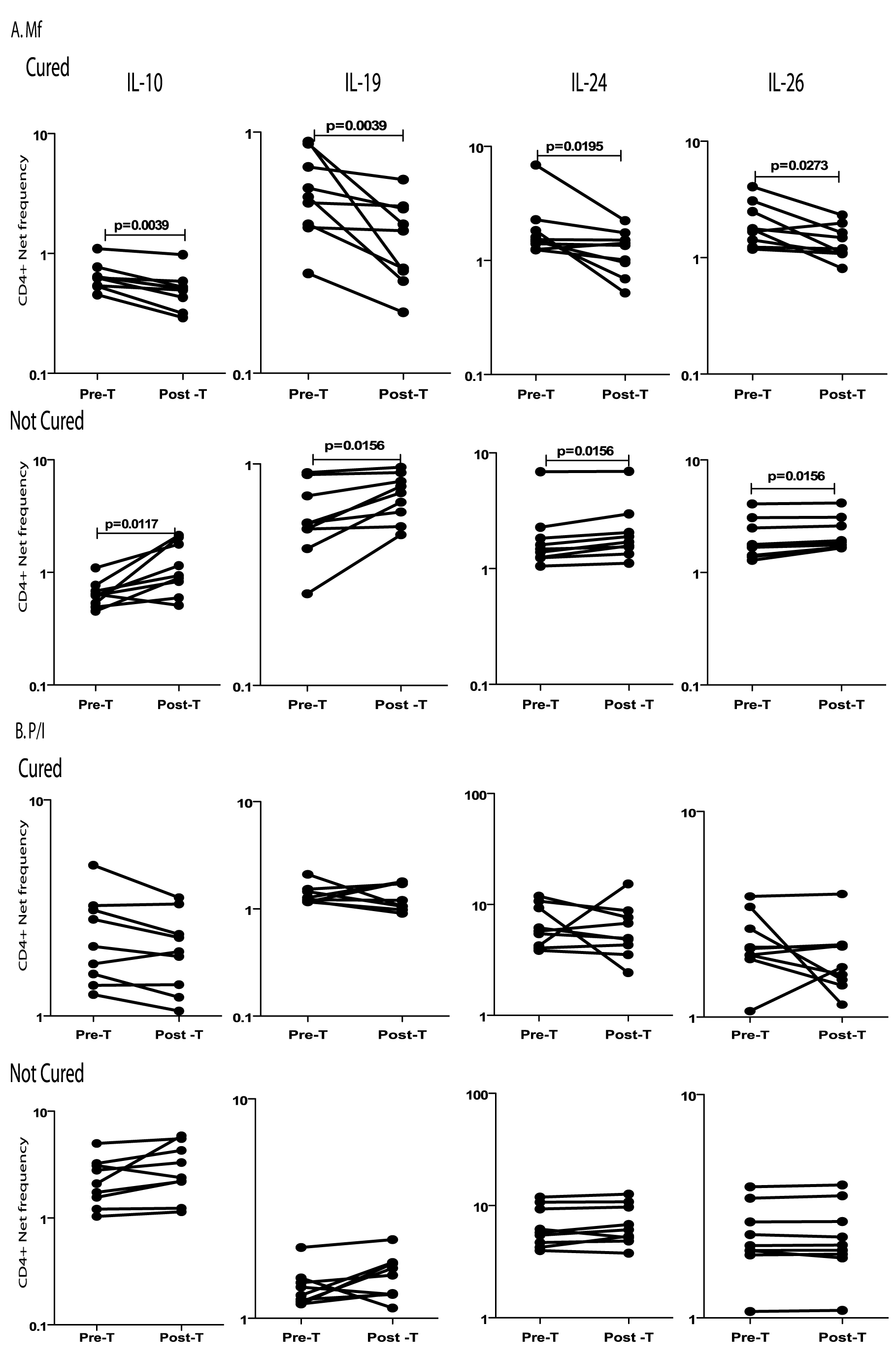

Supplement: Figure S1 — Treatment of filarial infection is associated with alterations in the frequency of CD4+ T cells expressing IL-10, IL-19, IL-24 and IL-26. The frequencies of CD4+ T cells expressing IL-10, IL-19, IL-24 and IL-26 following stimulation with Mf (A) or PMA/ionomycin (B) before and after treatment with a standard dose of DEC and albendazole in a subset of INF individuals (Cured, n = 9), who turned circulating antigen negative and another set of INF individuals (Not cured, n = 7), who remained circulating antigen positive. Antigen – stimulated frequencies are shown as net frequencies with the baseline levels subtracted. Each line represents a single individual. P values were calculated using the Wilcoxon signed rank test. (TIF) [file pntd.0002762.s001.tif]

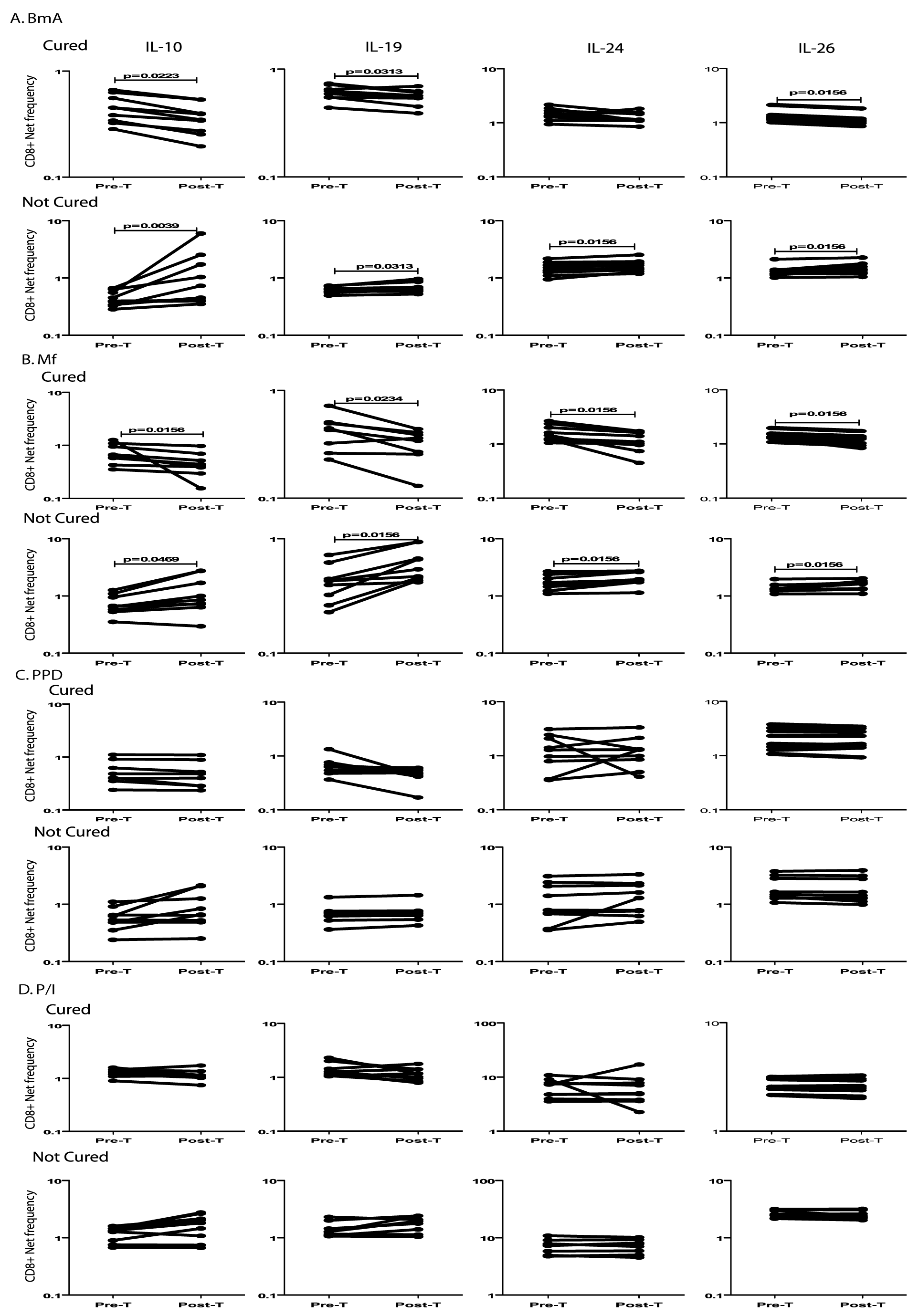

Supplement: Figure S2 — Treatment of filarial infection is associated with alterations in the frequency of CD8+ T cells expressing IL-10, IL-19, IL-24 and IL-26. The frequencies of CD8+ T cells expressing IL-10, IL-19, IL-24 and IL-26 following stimulation with BmA (A), Mf (B), PPD (C) and PMA/ionomycin (D) before and after treatment with a standard dose of DEC and albendazole in a subset of INF individuals (Cured, n = 9), who turned circulating antigen negative and another set of INF individuals (Not cured, n = 7), who remained circulating antigen positive. Antigen – stimulated frequencies are shown as net frequencies with the baseline levels subtracted. Each line represents a single individual. P values were calculated using the Wilcoxon signed rank test. (TIF) [file pntd.0002762.s002.tif]
